# Copper and zinc hydroxychloride cosupplementation improve growth performance and carcass and reduce diarrhea frequency in grower-finisher pigs

**DOI:** 10.1093/tas/txab202

**Published:** 2021-11-15

**Authors:** Maitê Vidal Mendonça, Denis Hideki Nakasone, Cristian Hernando Garcia Martinez, Jéssica Luana Gemelli, Angélica Simone Cravo Pereira, Silvana Marina Piccoli Pugine, Mariza Pires de Melo, André Furugen Cesar de Andrade, Lúcio Francelino Araújo, Karolina Von Zuben Augusto, Han Yanming, Simone Maria Massami Kitamura Martins

**Affiliations:** 1 Department of Nutrition and Animal Production, FMVZ, University of São Paulo, Pirassununga, SP 13635-900, Brazil; 2 Department of Basic Sciences, FZEA, University of Sao Paulo, Pirassununga, SP 13635-900, Brazil; 3 Department of Animal Science, FZEA, University of Sao Paulo, Pirassununga, SP 13635-900, Brazil; 4 Trouw Nutrition R&D, 3800 AG Amersfoort, Utrecht, the Netherlands

**Keywords:** antioxidant capacity, fecal score, inorganic minerals, pork meat quality, swine

## Abstract

This study investigated copper (Cu) and zinc (Zn) hydroxychloride cosupplementation on the growth performance, diarrhea frequency, carcass, meat quality, and antioxidant activity in grower-finisher pigs. A total of 256 pigs were used from 70 to 154 days (d) of age, distributed in four treatments, with eight pigs in each pen and eight replications per treatment. Diets were provided to grower pigs from 70 to 112 days old and in the finisher, 112 to 154 days old. Copper was considered the low level at 100 mg Cu/kg and 90 mg Cu/kg, respectively, and 150 mg Cu/kg in both periods as high in the grower and finisher periods. In the grower and finisher period, zinc was cosupplemented in the diet at 80 mg Zn/kg and 70 mg Zn/kg, respectively. In the diets, T1 and T2 groups are the traditional inorganic sources for minerals (copper sulfate, CuSO_4_; zinc oxide, ZnO) and T3 and T4 hydroxychloride sources (copper hydroxychloride, CHC, and zinc hydroxychloride, ZHC). The flavomycin was associated with treatments with low Cu content in the inclusion of 50 g/ton. The experimental design was in randomized blocks, the data were submitted to analysis of PROC MIXED in SAS, the PDIFF test analyzed the treatment effect. At the finisher period, pigs fed both minerals from hydroxychloride source had a higher BW 154 d, average daily gain (ADG) 70 to 154 d, the hot and cold carcass weight and frequency of normal feces than those fed 150 mg Cu/kg and Zn from a traditional inorganic source (*P* < 0.05). The animals fed low Cu levels of the sulfate source had a higher ADG 70 to 154 d than those fed high Cu levels of the same source (*P* < 0.05). Pigs fed 150 mg Cu/kg cosupplemented with Zn from a hydroxychloride source had the highest carcass length (*P* < 0.05). There was no difference among the treatments for meat quality (*P* > 0.05). Pigs fed 150 mg Cu/kg and Zn from a traditional inorganic source had a higher superoxide dismutase (SOD) activity than the other treatments (*P* < 0.05). Animals fed low Cu levels from hydroxychloride had a higher malondialdehyde (MDA) formation than those fed sulfate source, regardless of the Cu levels and those fed high Cu levels of hydroxychloride (*P* < 0.05). In conclusion, 150 mg Cu/kg as copper sulfate cosupplemented to zinc oxide in the diet of growing and finishing pigs impairs the growth performance, carcass and increases diarrhea frequency, and copper and zinc hydroxychloride cosupplementation improves these characteristics.

## INTRODUCTION

It is predicted that the global economy will continue to grow, and pork meat demand will increase in most countries. Thus, pig productions must obtain improvements in production rates. Among the ways to improve productive indexes are advances in management, genetic improvement, animal welfare, sanitary conditions, and nutrition. Among the several alternatives to be used in pig nutrition, minerals in the diet stand out.

Amidst the minerals, copper (Cu) and zinc (Zn) have aroused more interest, due to both minerals at a pharmacological level can act as antimicrobial (Pérez et al., 2011), growth promoters (Zhang and Guo, 2007; Coble et al., 2017), reduce diarrhea (Zhang and Guo, 2007), can act as cofactors of antioxidants enzymes (Kim et al., 2018).

The hydroxychloride mineral source has a crystalline structure formed by covalent bonds between the soluble metal ion, multiple hydroxyl groups, and chloride ions (Cemin et al., 2017). Besides, the low solubility in water and high solubility in acid solutions (Cao et al., 2000) makes them less reactive with other diet components (Olukosi et al., 2018). Thus, this mineral compound offers more stability in the feed and digestive tract and greater bioavailability, resulting in benefits on growth performance and pork meat quality than traditional inorganic sources (sulfate and oxide) (Zhang and Guo, 2007).

Few studies evaluated carcass characteristics, meat quality, and antioxidant capacity in pigs fed Cu e Zn cosupplemented at a high level and from a hydroxychloride source. Combining these hydroxychloride minerals does not constantly improve growth performance and carcass characteristics when pigs are fed 125 mg Cu/kg and 150 mg Zn/kg (Feldpausch et al., 2016). The effect of minerals varies depending on the age, level and source, immune status, type of ingredients, and interaction of microelements, which may affect other nutrients’ absorption and bioavailability.

There were more studies with these minerals separately, in which pigs fed 150 mg Cu/kg had an improved growth performance (Coble et al., 2017; Espinosa et al., 2020), but no difference was observed with 130 mg Cu/kg (Zheng et al., 2018). Similarly, Zn showed an improved growth performance when pigs were fed 80 to 100 mg Zn/kg (Carpenter et al., 2016; Van Kuijk et al., 2019), and with 150 mg increase in the percentage of carcass yield was observed (Cemin et al., 2019).

Based on the literature, we hypothesized that due to the mineral sources’ chemical properties, the growth performance and carcass quality of grower-finisher pigs fed Cu and Zn hydroxychloride minerals would be higher than those fed inorganic sources. Therefore, the present experiment was conducted to evaluate the copper hydroxychloride (CHC) and zinc hydroxychloride (ZHC) cosupplementation in the diet of growing and finishing pigs on performance, diarrhea frequency, carcass, meat quality, and antioxidant capacity.

## MATERIALS AND METHODS

The sampling procedures, animal care, and experimental use of animals were approved by the Ethics Committee on Animal Use of the School of Veterinary Medicine and Animal Science (CEUA/FMVZ) at the University of Sao Paulo with the rules issued by the National Council for Control of Animal Experimentation (CONCEA), protocol number 4110030918. The trial was performed in the Swine Farm from USP *Campus* “Fernando Costa” at the University of Sao Paulo in Pirassununga.

### Animals, Experimental Design, and Diets

A total of 256 commercial crossbred [(Large White × Landrace) × Duroc] pigs at 70 days of age were used in an 84-day feeding trial. Upon entry into the facility, pigs were individually weighed, identified with a unique ear tag, and allotted one of four treatments based on body weight (BW) (27.70 ± 0.33 kg) in a randomized block design experiment. Each pen was equipped with a concrete floor, providing 1.25 m^2^/animal, concrete feed troughs, and a nipple drinker with ad libitum access to feed and water. The lighting and ambient temperature were natural.

The experimental diets were isoproteic and isoenergetic, and the nutrient contents were based on the recommendations of NRC (2012) in Table 1. Each treatment consisted of 8 replicate pens with 8 animals each, totalizing 64 pigs per treatment. The diets were provided during the growing (period 1, from 70 to 91 days old; period 2, from 91 to 112 days old) and the finishing period from 112 to 154 days old. The formulation of these diets was similar for all treatments.

**Table 1.** Ingredients and calculated composition of experimental diets (as-fed basis)^1^

Item	Grower 1				Grower 2				Finisher			
	T1	T2	T3	T4	T1	T2	T3	T4	T1	T2	T3	T4
Ingredient, %												
Corn	63.95	63.93	63.97	63.96	68.95	68.93	68.97	68.96	75.95	75.93	75.97	75.96
Soybean meal	32.00	32.00	32.00	32.00	28.00	28.00	28.00	28.00	22.00	22.00	22.00	22.00
Bnubi VR 12000^2^	4.00	4.00	4.00	4.00	–	–	–	–	–	–	–	–
Bnubi VR 13000^3^	–	–	–	–	3.00	3.00	3.00	3.00	–	–	–	–
Bnubi VR 14000^4^	–	–	–	–	–	–	–	–	2.00	2.00	2.00	2.00
CuSO_4_	0.040	0.060	–	–	0.040	0.060	–	–	0.036	0.060	–	–
ZnO	0.011	0.011	–	–	0.011	0.011	–	–	0.010	0.010	–	–
CHC	–	–	0.019	0.028	–	–	0.019	0.028	–	–	0.017	0.028
ZHC	–	–	0.015	0.015	–	–	0.015	0.015	–	–	0.013	0.013
Calculated composition^5^												
Crude protein, %	19.97	19.97	19.97	19.97	18.40	18.40	18.40	18.40	16.20	16.20	16.20	16.20
ME, kcal/kg	3,141	3,141	3,141	3,141	3,180	3,180	3,180	3,180	3,233	3,233	3,233	3,233
Lysine, %	1.150	1.150	1.150	1.150	0.960	0.960	0.960	0.960	0.800	0.800	0.800	0.800
Calcium, %	0.816	0.816	0.816	0.816	0.702	0.702	0.702	0.702	0.690	0.690	0.690	0.690
Phosphorus, %	0.654	0.654	0.654	0.654	0.567	0.567	0.567	0.567	0.525	0.525	0.525	0.525
Copper, mg/kg	100.00	150.00	100.00	150.00	100.00	150.00	100.00	150.00	90.00	150.00	90.00	150.00
Zinc, mg/kg	80.00	80.00	80.00	80.00	80.00	80.00	80.00	80.00	70.00	70.00	70.00	70.00

^1^All pigs received allotted treatment diet upon starting 70 days of age. Treatments: Grower *1 (70-91 d) and Grower 2 (91-112 d)*, T1: 100 mg Cu/kg as CuSO_4_; T2: 150 mg Cu/kg as CuSO_4_ and both with 80 mg Zn/kg as ZnO; T3: 100 mg Cu/kg as CHC and T4: 150 mg Cu/kg as CHC and both with 80 mg Zn/kg as ZHC; Finisher (112 to 154 d), T1: 90 mg Cu/kg as CuSO_4_; T2: 150 mg Cu/kg as CuSO_4_ and 70 mg Zn/kg as ZnO in both treatments; T3: 90 mg Cu/kg as CHC; T4: 150 mg Cu/kg as CHC and both with 70 mg Zn/kgasZHC. T1 and T3 = Flavomycin inclusion (50 g/ ton.).

^2^Bnubi VR 12000^®^ (per kg of product): calcium 155 g, phosphorus 36 g, fluorine 360 mg, sodium 48 g, methionine 980 mg, lysine 15.5 g, threonine 985 mg, choline 800 mg, cobalt 10 mg, iron 2,000 mg, iodine 25 mg, manganese 875 mg, selenium 11.25 mg, vitamin A 250,000 UI, vitamin D3 50,000 UI, vitamin E 625 UI, vitamin K3 55 mg, vitamin B1 50 mg, vitamin B2 112.5 mg, vitamin B6 62.5 mg, vitamin B12 625 mcg, niacin 750 mg, pantothenic acid 450 mg, folic acid 62.5 mg, biotin 6.25 mg, B.H.T 2,500 mg, 6-phytase 25,000 ftu, endo-1,4-beta xylanase 288,125 u).

^3^Bnubi VR 13000^®^ (per kg of product): calcium 155 g, phosphorus 23,1 g, fluorine 231 mg, sodium 65 g, choline 582,5 mg, cobalt 10,667 mg, iron 2.133,333 mg, iodine 26,667 mg, manganese 933,333 mg, selenium 12 mg, vitamin A 133,333.333 UI, vitamin D3 33.333,333 UI, vitamin E 413,333 UI, vitamin K3 66,667 mg, vitamin B1 40 mg, vitamin B2 113,333 mg, vitamin B6 66,667 mg, vitamin B12 583,333 mcg, niacin 583,333 mg, pantothenic acid 333,333 mg, folic acid 10 mg, biotin 2,667 mg, B.H.T 2,000 mg, 6-phytase 33,333.333 u), flavomycin 133,333 mg or without flavomycin.

^4^Bnubi VR 14000^®^ (per kg of product): calcium 235 g, phosphorus 23.125 g, fluorine 231.25 mg, sodium 97.67 g, choline 625.5 mg, cobalt 14 mg, iron 2,800 mg, iodine 35 mg, manganese 1,225 mg, selenium 15.75 mg, vitamin A 150,000 UI, vitamin D3 37,500 UI, vitamin E 465 UI, vitamin K3 75 mg, vitamin B1 45 mg, vitamin B2 127.5 mg, vitamin B6 75 mg, vitamin B12 656.25 mcg, niacin 656.25 mg, pantothenic acid 375 mg, folic acid 11.3 mg, biotin 3 mg, B.H.T 3,000 mg, 6-phytase 25,000 u, flavomycin 200 mg or without flavomycin.

^5^ME = Metabolizable energy.

In the grower and finisher diet, 100 mg Cu/kg and 90 mg Cu/kg diet were considered low, and 150 mg Cu/kg in both diets was high. Zinc was cosupplemented in the diet in 80 mg Zn/kg in grower and 70 mg Zn/kg diet in finisher diet. The treatments T1 and T2 animals received the traditional inorganic source for both minerals (copper sulfate, CuSO_4_ and zinc oxide, ZnO), and T3 and T4 received the hydroxychloride source (CHC and ZHC). The flavomycin was associated with treatments with low Cu levels in the inclusion of 50 g/ton in the three diets. In the grower 1 diet, flavomycin and Cu and Zn were mixed with a small amount of corn as the carrier and then added to a mixer to prepare the diet. In the diet of grower 2 and finisher, only Cu and Zn were mixed with corn as the carrier. Bnubi VR 13000 and 14000 premixes, used in the grower 2 and finisher diets, respectively, were produced with or without the addition of flavomycin. Copper and zinc were supplemented by replacing the same amount of corn in different phases’ basal diet.

### Growth Performance

Individual BW was evaluated on days 70, 84, 91, 98, 112, 126, 140, and 154, and the average daily gain (ADG), average daily feed intake (ADFI), and feed conversion ratio (FCR) in the intervals between them.

### Diarrhea Frequency

The fecal score was observed daily from 70 to 154 days of age and classified as: 1) solid stools (considered as normal feces); 2) stools less consistent than normal (considered as pasty feces), and 3) liquid stools (considered as severe diarrhea) according to Pascoal et al. (2012). The classification was considered when two or more animals in pen showed the highest score. Subsequently, it was calculated the diarrhea incidence, considering diarrhea the sum of scores 2 and 3.

### Carcass Performance

At 154 days, at the end of the experiment, the pigs were slaughtered by incision of the jugular vein after being stunned by electric shock (high voltage, low current). It was measured the hot carcass weight and the cold carcass weight after 24 h. After 24 h of cooling, the carcass’s straight length (cm) was measured from the forward edge of the first rib to the forward edge of the pubic symphysis using a measuring tape. Average backfat thickness (mm) was obtained by averaging the three measures of backfat opposite the first rib, last rib, and last lumbar vertebra, using a digital caliper (Absolute Digimatic, model CD-8“ CX-B, Mitutoyo Sul Americana Ltda. São Paulo, Brazil) (Ray, 2004).

### Meat Quality

Among the 256 animals, 10 animals per treatment were randomly selected for meat quality evaluation, totalizing 40 animals. The temperature and pH were measured at 1 h and 24 h post-mortem, according to Chen et al. (2010). Briefly, both were measured in Semimembranosus muscle using a portable digital pH meter with insertion electrode (Hanna Instruments Inc, model HI 99163, Woonsocket, RI, USA), and the pH meter was adjusted using standard liquids before usage (pH = 4 and 7).

The loin eye area was measured in the *Longissimus dorsi* muscle between the 11th to the 13th rib from the left side carcass (Li et al., 2015) after 24 h of cooling. It was used a grid ruler with scale in square centimeters (0.5 cm^2^).

After 24 h of slaughter, the color was measured in the sample of 2.5 cm thick using a portable colorimeter (MiniScan EZ, model CM2500d, Minolta Camera Co. Ltd., Osaka, Japan) after exposition to oxygen for 20 min at room temperature, setting on the L* (brightness), a* (red-green component), and b* (yellow-blue component), according to the CIELAB system (1986). The chroma (meat saturation) and the hue were calculated (Teye et al., 2006).

The samples used to evaluate the cooking loss and shear force were vacuum packed in polyethylene bags (Cryovac, Charlotte, NC, USA) and kept in a freezer at −20 °C until all samples were collected. The cooking loss was measured according to Honikel (1998). The sample was thawed in a refrigerator at approximately 7 °C for 24 h. Then, it was weighed and cooked at 170 °C until it reached an internal temperature of 71 °C, without adding season (AMSA, 2015). After it cooled was weighed again. The internal temperature of the loin was monitored using digital perforation thermometers inserted in the samples to their geometric center.

According to Wheeler et al. (2005), the shear force was measured considering the average values obtained among six cylinders. The cylinders were sheared on a Brookfield CT-3 Texture Analyzer (Brookfield, USA) texturometer equipped with a Warner-Bratzler Shear blade for shear force determination.

Drip loss of loin was measured by the gravimetric method according to Honikel (1998), and the results were expressed as a percentage of weight loss. Briefly, the sample of 2 cm thick (approximately 100 g) was weighed and suspended in a bag for 48 h in the cold room at 2 to 4 °C, ensuring that the meat does not touch the sides of the bag. Then, the meat was weighed again.

The metmyoglobin was measured in fresh *Longissimus dorsi* muscle using the spectrophotometer (Beckman Coulter model DU800), and readings were taken at the wavelengths of 525, 503, 557, and 582 nm. Analyzes were calculated according to the equations proposed by Krzywicki (1979), Tang et al. (2004), and Lins et al. (2017) to evaluate the concentrations of oxymyoglobin and metmyoglobin.

### Antioxidant Capacity and Determination of Thiobarbituric Acid Reactive Substances (TBARS)

The superoxide dismutase (SOD) activity was determined in *Longissimus dorsi* according to Ewing and Janero (1995) with modifications. The SOD activity was determined according to the rate of nitro blue tetrazolium (NBT) reduction by superoxide anion at 25 °C for 5 min in a spectrophotometer (Thermo Scientific Multiskan FC) at 540 nm absorbance. The protein content of each sample was determined by the Bradford (1976) method, and the albumin calibration curve was used to obtain the standard curve. The SOD activity was expressed in U/mg protein.

Glutathione peroxidase (GSH-Px) and catalase (CAT) enzymes were measured in *Longissimus dorsi* according to Paglia and Valentine (1967) and Beers and Sizer (1952), respectively with modifications. The maximum activity of GSH-Px was evaluated by decreasing nicotinamide adenine dinucleotide phosphate (NADPH) concentration at 37 °C for 3 min by spectrophotometry (Beckman Coulter model DU800) reading at 340 nm. The protein content of each sample was determined by the Bradford (1976) method and albumin calibration curve was used to obtain the standard curve. Results were expressed in U/g protein.

The catalase activity was determined according to Natori et al. (2019). The reduction rate of hydrogen peroxide (H_2_O_2_) in H_2_O and O_2_. The H_2_O_2_ consumption was read at 240 nm using a DU-800 spectrophotometer (Beckman Coulter, Brea, CA, USA) for 3 min at 25 °C.

Lipid oxidation was measured in duplicate by thiobarbituric acid reactive substances (TBARS), according to Lins et al. (2017). The absorbance of the samples was read at 532 nm and 600 nm by spectrophotometer (Beckman Coulter model DU800), and the difference between these absorbances corrected the sample turbidity. The standard curve obtained from 1,1,3,3-tetraethoxypropane (TEP) solutions from 0.02 to 1.2 μg/mL. Results were expressed as milligrams of malondialdehyde (MDA) per kg of meat.

### Statistical Analysis

Data were submitted to analysis of variance (PROC MIXED) using the procedure of SAS Enterprise Guide (version 9.4) (SAS Institute, Inc., Cary, NC), with previous verification of the normality of the residues and homogeneity of the variances. The design was in randomized blocks with repeated measures. The treatment and days were considered a fixed effect, and the batch was considered a random effect. The experimental unit was the pen with eight animals each for ADFI, FCR, and diarrhea frequency. For the other variables, the experimental unit was the animal. For growth performance, carcass and meat quality was considered the BW initial as a covariate, for capacity oxidant was considered pH 1h as a covariate. Values of diarrhea frequency were transformed into arcsine, and the PDIFF test analyzed the difference between the means of the treatments. The significance level considered was *P* < 0.05, and *P* ≤ 0.10 was considered a trend for testing the main effects. All results were expressed as mean ± standard error of the mean (SEM).

## RESULTS

### Growth Performance

**Figure 1.**
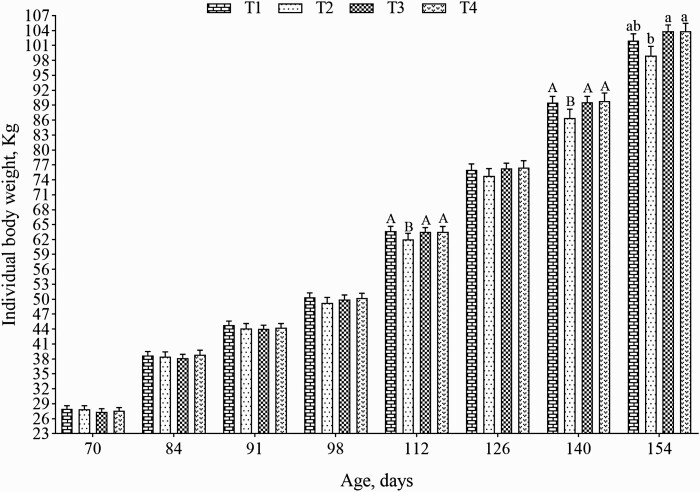
Individual body weight of pigs from 70 to 154 days of age.^1^ ^a,b^Different lowercase letters are significantly different (PDIFF, *P* < 0.05). ^A,B^Different capital letters are trend (PDIFF, *P* > 0.05). ^1^Values represent mean ± standard error of the means of 64 pigs. Treatments: Grower 1 (70 to 91 d) and Grower 2 (91 to 112 d), T1: 100 mg Cu/kg as CuSO_4_; T2: 150 mg Cu/kg as CuSO_4_ and both with 80 mg Zn/kg as ZnO; T3: 100 mg Cu/kg as CHC and T4: 150 mg Cu/kg as CHC and both with 80 mg Zn/kg as ZHC; Finisher (112 to 154 d), T1: 90 mg Cu/kg as CuSO_4_; T2: 150 mg Cu/kg as CuSO_4_ and 70 mg Zn/kg as ZnO in both treatments; T3: 90 mg Cu/kg as CHC; T4: 150 mg Cu/kg as CHC and both with 70 mg Zn/kg as ZHC. T1 and T3 = Flavomycin inclusion (50 g/ton.).

There was no interaction between treatments and days (*P* > 0.05) for any performance characteristics. At the end of the grower period, animals fed both minerals from hydroxychloride source (T3 and T4) tended to have a higher ADG 70 to 112 d than those fed 150 mg Cu/kg as CuSO_4_ cosupplemented with zinc oxide, and they also were similar to those that received 100 mg Cu/kg and Zn of the traditional inorganic mineral (*P* = 0.051; Table 2). At finisher period, pigs fed Cu and Zn from hydroxychloride source (T3 and T4) had a higher BW 154 d and ADG 70 to 154 d than those fed 150 mg Cu/kg and zinc of the traditional inorganic source (*P* < 0.05; T2), and it also showed similar BW those fed 90 mg Cu/kg from CuSO_4_ and zinc oxide (*P* < 0.05; Figure 1, Table 2). No differences were observed on BW 154 d between the pigs that received traditional inorganic sources (T1 and T2). The animals fed low Cu level and Zn of the traditional inorganic source had similar ADG 70 to 154 d to those fed both minerals from hydroxychloride source, regardless of the Cu level. Besides, those fed 150 mg Cu/kg of copper sulfate and zinc oxide had the lowest ADG 70 to 154 d (*P* < 0.05; Table 2). At the end of the finisher period, animals fed both minerals from hydroxychloride sources (T3 and T4) tended to have a greater ADFI 140 to 154 d than those fed 150 mg Cu/kg and Zn of traditional inorganic (T2), and they also were similar to those fed 90 mg Cu/kg from CuSO_4_ (T1). No differences were observed between the animals that received the hydroxychloride inorganic mineral source (*P* = 0.086; Table 2). Concerning the FCR, the treatments provided to the pigs did not influence these characteristics (*P* > 0.05; Table 2).

**Table 2.** Growth performance of pigs from 70 to 154 days of age^1^

Item	Treatment				*P*-value
	T1	T2	T3	T4	
Average daily gain, kg/d					
70–84 days	0.76 ± 0.03	0.76 ± 0.03	0.79 ± 0.03	0.81 ± 0.03	0.618
84–91 days	0.88 ± 0.03	0.80 ± 0.05	0.81 ± 0.06	0.82 ± 0.04	0.624
91–98 days	0.79 ± 0.04	0.72 ± 0.04	0.83 ± 0.04	0.82 ± 0.03	0.261
98–112 days	0.95 ± 0.02	0.91 ± 0.02	0.97 ± 0.02	0.95 ± 0.03	0.489
70–112 days	0.85 ± 0.01^A^	0.81 ± 0.02^B^	0.86 ± 0.01^A^	0.85 ± 0.02^A^	0.051
112–126 days	0.88 ± 0.03	0.88 ± 0.02	0.91 ± 0.03	0.93 ± 0.03	0.155
126–140 days	0.97 ± 0.03	0.88 ± 0.03	0.95 ± 0.03	1.00 ± 0.04	0.186
140–154 days	0.92 ± 0.03	0.88 ± 0.02	0.99 ± 0.03	0.97 ± 0.02	0.195
70–154 days	0.88 ± 0.01^a^	0.85 ± 0.01^b^	0.91 ± 0.01^a^	0.91 ± 0.01^a^	0.001
Average daily feed intake, kg/d					
70–84 days	1.59 ± 0.07	1.57 ± 0.09	1.59 ± 0.08	1.58 ± 0.10	0.915
84–91 days	1.94 ± 0.06	1.84 ± 0.09	1.81 ± 0.09	1.89 ± 0.08	0.511
91–98 days	2.06 ± 0.06	1.97 ± 0.08	2.01 ± 0.08	2.07 ± 0.10	0.658
98–112 days	2.37 ± 0.08	2.29 ± 0.11	2.42 ± 0.06	2.40 ± 0.11	0.392
70–112 days	1.99 ± 0.06	1.92 ± 0.09	1.95 ± 0.07	1.98 ± 0.09	0.643
112–126 days	2.68 ± 0.11	2.59 ± 0.12	2.78 ± 0.07	2.75 ± 0.13	0.391
126–140 days	2.83 ± 0.08	2.87 ± 0.13	2.90 ± 0.08	3.01 ± 0.13	0.687
140–154 days	3.22 ± 0.08^AB^	3.07 ± 0.10^B^	3.34 ± 0.06^A^	3.29 ± 0.13^A^	0.086
70–154 days	2.38 ± 0.06	2.32 ± 0.09	2.41 ± 0.06	2.43 ± 0.10	0.473
Feed conversion ratio, kg/kg					
70–84 days	2.20 ± 0.24	2.16 ± 0.17	2.17 ± 0.24	2.10 ± 0.24	0.911
84–91 days	2.31 ± 0.18	2.45 ± 0.23	2.50 ± 0.23	2.59 ± 0.28	0.570
91–98 days	2.66 ± 0.13	2.79 ± 0.20	2.54 ± 0.15	2.60 ± 0.18	0.556
98–112 days	2.50 ± 0.11	2.53 ± 0.09	2.38 ± 0.13	2.54 ± 0.11	0.973
70–112 days	2.35 ± 0.08	2.36 ± 0.06	2.28 ± 0.10	2.32 ± 0.07	0.761
112–126 days	3.10 ± 0.15	2.95 ± 0.07	3.10 ± 0.12	3.03 ± 0.20	0.960
126–140 days	3.00 ± 0.21	3.33 ± 0.12	3.01 ± 0.10	3.01 ± 0.08	0.280
140–154 days	3.68 ± 0.18	3.48 ± 0.17	3.38 ± 0.19	3.57 ± 0.21	0.305
70–154 days	2.71 ± 0.05	2.73 ± 0.04	2.65 ± 0.08	2.69 ± 0.06	0.601

^a,b^Different lowercase letters are significantly different (PDIFF, *P* < 0.05).

^A,B^Different capital letters are trend (PDIFF, *P* ≤ 0.10).

^1^Values represent mean ± standard error of the means of 8 pens with 8 pigs. Treatments: Grower 1 (70 to91 d) and Grower 2 (91 to 112 d), T1: 100 mg Cu/kg as CuSO_4_; T2: 150 mg Cu/kg as CuSO_4_ and both with 80 mg Zn/kg as ZnO; T3: 100 mg Cu/kg as CHC and T4: 150 mg Cu/kg as CHC and both with 80 mg Zn/kg as ZHC; Finisher (112to 154 d), T1: 90 mg Cu/kg as CuSO_4_; T2: 150 mg Cu/kg as CuSO_4_ and 70 mg Zn/kg as ZnO in both treatments; T3: 90 mg Cu/kg as CHC; T4: 150 mg Cu/kg as CHC and both with 70 mg Zn/kg as ZHC. T1 and T3 = Flavomycin inclusion (50 g/ton.).

### Diarrhea Frequency

**Figure 2.**
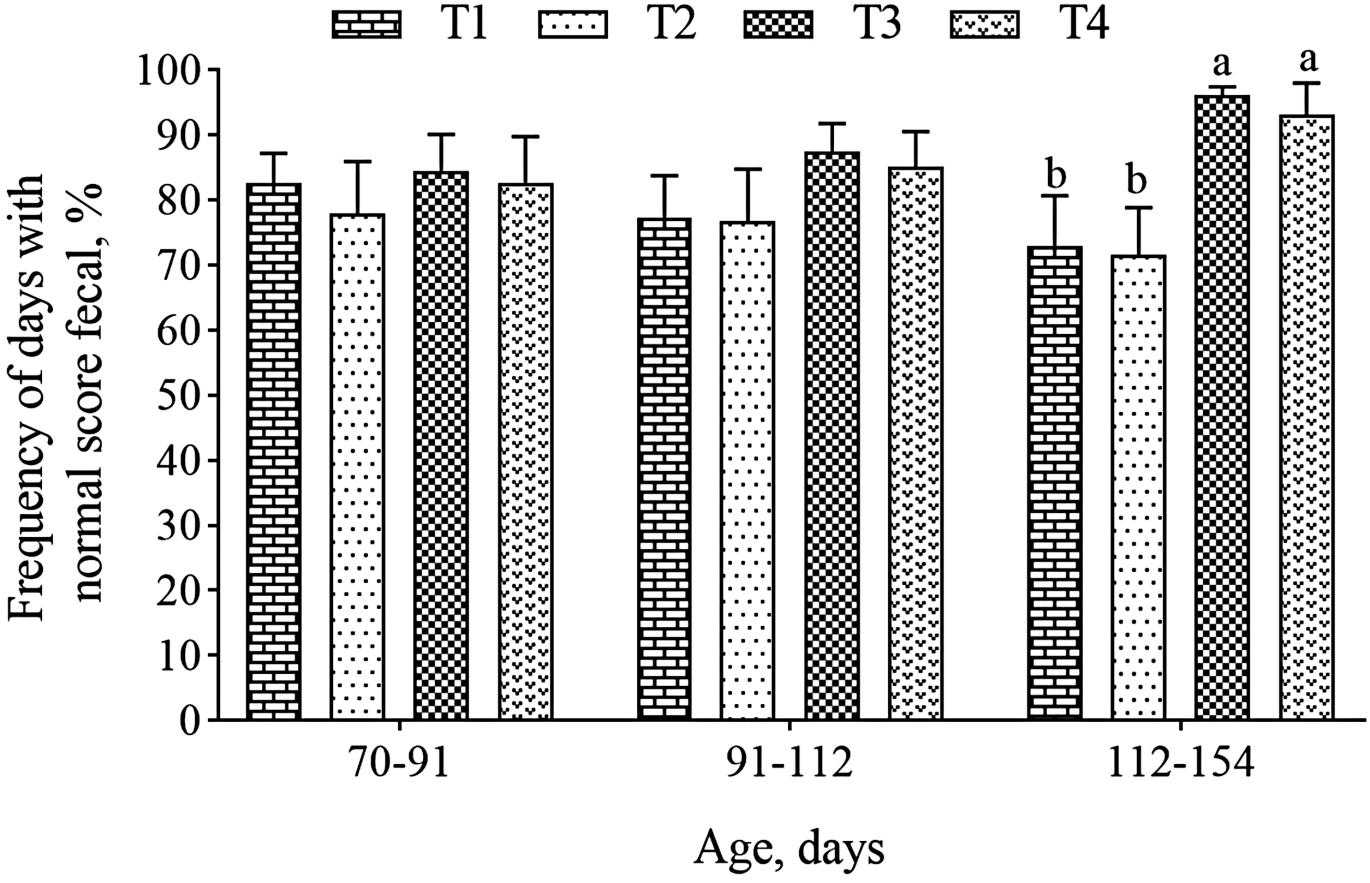
Frequency of normal score fecal.^1^ ^a,b^Different lowercase letters are significantly different (PDIFF, *P* < 0.05). ^1^Values represent mean ± standard error of the means of 8 pens with 8 pigs. Treatments: Grower 1 (70 to 91 d) and Grower 2 (91 to 112 d), T1: 100 mg Cu/kg as CuSO_4_; T2: 150 mg Cu/kg as CuSO_4_ and both with 80 mg Zn/kg as ZnO; T3: 100 mg Cu/kg as CHC and T4: 150 mg Cu/kg as CHC and both with 80 mg Zn/kg as ZHC; Finisher (112 to 154 d), T1: 90 mg Cu/kg as CuSO_4_; T2: 150 mg Cu/kg as CuSO_4_ and 70 mg Zn/kg as ZnO in both treatments; T3: 90 mg Cu/kg as CHC; T4: 150 mg Cu/kg as CHC and both with 70 mg Zn/kg as ZHC. T1 and T3 = Flavomycin inclusion (50 g/ton.).

There was no interaction for diarrhea frequency between treatments and days (*P* > 0.05). The pigs fed Cu and Zn from hydroxychloride sources (T3 and T4) showed a higher frequency of normal feces than those fed traditional inorganic sources (T1 and T2), regardless of Cu level (*P* < 0.05; Figure 2).

### Carcass Performance

At the end of the finisher period, pigs fed both minerals from hydroxychloride source (T3 and T4) had a greater hot and cold carcass weight than those fed 150 mg Cu/kg and Zn of the traditional inorganic source (T2) (*P* < 0.05; Table 3), but they also were similar to those fed 90 mg Cu/kg as CuSO_4_ and zinc oxide (T1) (*P* > 0.05; Table 3). Animals that received 150 mg Cu/kg and Zn from hydroxychloride source (T4) had the highest carcass length, and the other treatments were similar to each other (*P* < 0.05; Table 3). No differences were observed among the treatments concerning backfat, carcass yield, and meat yield (*P* > 0.05; Table 3).

**Table 3.** Values of carcass performance at 154 days of age^1^

Item	Treatment				*P*-value
	T1	T2	T3	T4	
Hot carcass weight, kg	77.86 ± 1.12^a^	75.73 ± 1.48^b^	78.99 ± 0.98^a^	78.60 ± 1.37^a^	<0.001
Cold carcass weight, kg	76.12 ± 1.09^a^	73.99 ± 1.44^b^	77.19 ± 0.96^a^	76.72 ± 1.34^a^	0.004
Carcass length, cm	78.69 ± 0.42^b^	78.37 ± 0.62^b^	79.12 ± 0.39^b^	80.03 ± 0.49^a^	0.002
Backfat thickness, mm	21.07 ± 0.60	20.62 ± 0.62	21.06 ± 0.46	21.04 ± 0.59	0.918
Carcass yield, %	76.41 ± 0.32	76.41 ± 0.23	76.01 ± 0.23	76.11 ± 0.25	0.953
Meat yield, %	59.10 ± 0.82	60.11 ± 0.80	60.51 ± 0.37	59.91 ± 0.82	0.529

^a,b^Different lowercase letters are significantly different (PDIFF, *P* < 0.05).

^1^Values represent mean ± standard error of the means of 64 pigs. Treatments: Grower 1 (70 to 91 d) and Grower 2 (91 to 112 d), T1: 100 mg Cu/kg as CuSO_4_; T2: 150 mg Cu/kg as CuSO_4_ and both with 80 mg Zn/kg as ZnO; T3: 100 mg Cu/kg as CHC and T4: 150 mg Cu/kg as CHC and both with 80 mg Zn/kg as ZHC; Finisher (112to 154 d), T1: 90 mg Cu/kg as CuSO_4_; T2: 150 mg Cu/kg as CuSO_4_ and 70 mg Zn/kg as ZnO in both treatments; T3: 90 mg Cu/kg as CHC; T4: 150 mg Cu/kg as CHC and both with 70 mg Zn/kg as ZHC. T1 and T3 = Flavomycin inclusion (50 g/ton.).

### Meat Quality

Regarding the variables evaluated at 1 h post-mortem, there was no difference among the treatments received by pigs (*P* > 0.05; Table 4). The pigs fed 90 mg Cu/kg cosupplemented with Zn from hydroxychloride source (T3) tended to have a higher loin eye area than those that received both minerals from traditional inorganic source, regardless of Cu level (T1 and T2), and also those fed 150 mg Cu/kg and zinc of hydroxychloride source (T4) (*P* = 0.059; Table 4). The animals fed both minerals from traditional inorganic sources, regardless of the Cu level, tended to have a greater red intensity component (a*) in meat at 24 h post-mortem than those fed both minerals from hydroxychloride sources (*P* = 0.097; Table 4). Finally, pigs fed 150 mg Cu/kg and zinc of traditional inorganic source tended to have a higher percentage of cooking loss than those fed 90 mg Cu/kg, regardless of source, and they also were similar to pigs that received 150 mg Cu/kg cosupplemented with Zn from hydroxychloride source (*P* = 0.089; Table 4). Concerning the other variables related to meat quality, no difference was observed among the treatments (*P* > 0.05; Table 4).

**Table 4.** Values of meat quality at 154 days of age^1^

Item	Treatment				*P*-value
	T1	T2	T3	T4	
1 h post-slaughter					
Temperature, ºC	35.38 ± 0.51	36.73 ± 0.49	35.08 ± 0.55	34.70 ± 1.19	0.501
pH	6.06 ± 0.22	5.78 ± 0.24	6.11 ± 0.16	6.18 ± 0.25	0.157
Oxymyoglobin, %	58.49 ± 3.34	63.95 ± 7.60	58.16 ± 2.67	62.44 ± 8.13	0.696
Metmyoglobin, %	30.40 ± 2.31	32.11 ± 2.72	32.31 ± 2.67	32.26 ± 3.00	0.904
24 h post-slaughter					
Temperature, ºC	9.96 ± 0.66	10.37 ± 0.38	10.15 ± 0.52	9.92 ± 0.97	0.333
pH	5.66 ± 0.07	5.53 ± 0.08	5.53 ± 0.04	5.25 ± 0.09	0.471
Loin eye area, cm^2^	42.62 ± 1.63^B^	42.37 ± 2.39^B^	45.97 ± 0.84^A^	41.25 ± 1.03^B^	0.059
Color	25.87 ± 0.38	26.37 ± 0.95	25.56 ± 1.34	26.11 ± 0.18	0.438
L*	57.19 ± 0.93	58.11 ± 0.36	56.54 ± 0.32	58.29 ± 0.38	0.270
a*	6.69 ± 0.31^A^	6.79 ± 0.36^A^	6.35 ± 0.32^B^	6.22 ± 0.38^B^	0.097
b*	13.74 ± 0.13	14.20 ± 0.44	13.77 ± 0.27	13.80 ± 0.64	0.868
Chroma (saturation)	15.29 ± 0.20	15.75 ± 0.01	15.19 ± 0.02	15.15 ± 0.01	0.992
Hue	1.12 ± 0.02	1.13 ± 0.12	1.14 ± 0.48	1.15 ± 0.33	0.578
Drip loss, %	8.96 ± 1.15	9.71 ± 1.18	9.70 ± 0.99	10.44 ± 0.79	0.870
Cooking loss, %	25.25 ± 0.99^B^	28.74 ± 1.02^A^	25.75 ± 0.39^B^	26.08 ± 0.93^AB^	0.089
Shear force, kgf	5.28 ± 0.40	5.38 ± 0.28	5.20 ± 0.28	5.28 ± 0.40	0.982

^A,B^Different capital letters are trend (PDIFF, *P* ≤ 0.10.)

^1^Values represent mean ± standard error of the means of 10 pigs. Treatments: Grower 1 (70 to 91 d) and Grower 2 (91 to 112 d), T1: 100 mg Cu/kg as CuSO_4_; T2: 150 mg Cu/kg as CuSO_4_ and both with 80 mg Zn/kg as ZnO; T3: 100 mg Cu/kg as CHC and T4: 150 mg Cu/kg as CHC and both with 80 mg Zn/kg as ZHC; Finisher (112 to 154 d), T1: 90 mg Cu/kg as CuSO_4_; T2: 150 mg Cu/kg as CuSO_4_ and 70 mg Zn/kg as ZnO in both treatments; T3: 90 mg Cu/kg as CHC; T4: 150 mg Cu/kg as CHC and both with 70 mg Zn/kg as ZHC. T1 and T3 = Flavomycin inclusion (50 g/ton.).

### Antioxidant Capacity and Determination of TBARS

Pigs fed a 150 mg Cu/kg cosupplemented with Zn from a traditional inorganic mineral source had a higher SOD activity than the other treatments (*P* < 0.05; Table 5). Regarding TBARS, animals fed 90 mg Cu/kg and Zn from hydroxychloride sources had a higher MDA formation than those other treatments (*P* < 0.05; Table 5). There were no differences among the treatments concerning GSH-Px and CAT (*P* > 0.05; Table 5).

**Table 5.** Antioxidant capacity in pork meat at 154 days of age^1^

Item^2^	Treatment				*P*-value
	T1	T2	T3	T4	
SOD	17.61 ± 1.24^b^	23.45 ± 1.86^a^	19.22 ± 1.23^ab^	17.50 ± 1.31^b^	0.016
GPx	5.15 ± 0.79	4.82 ± 0.75	5.75 ± 0.86	4.86 ± 0.77	0.702
CAT	3438.39 ± 260.00	3814.36 ± 402.87	3620.32 ± 311.97	4164.40 ± 436.28	0.651
TBARS	0.12 ± 0.01^b^	0.13 ± 0.02^b^	0.18 ± 0.02^a^	0.13 ± 0.01^b^	0.019

^a,b^Different lowercase letters are significantly different (PDIFF, *P* < 0.05).

^1^Values represent mean ± standard error of the means of 10 pigs. Treatments: Grower 1 (70 to 91 d) and Grower 2 (91 to 112 d), T1: 100 mg Cu/kg as CuSO_4_; T2: 150 mg Cu/kg as CuSO_4_ and both with 80 mg Zn/kg as ZnO; T3: 100 mg Cu/kg as CHC and T4: 150 mg Cu/kg as CHC and both with 80 mg Zn/kg as ZHC; Finisher (112 to 154 d), T1: 90 mg Cu/kg as CuSO_4_; T2: 150 mg Cu/kg as CuSO_4_ and 70 mg Zn/kg as ZnO in both treatments; T3: 90 mg Cu/kg as CHC; T4: 150 mg Cu/kg as CHC and both with 70 mg Zn/kg as ZHC. T1 and T3 = Flavomycin inclusion (50 g/ton.).

^2^Abbreviations: SOD, superoxide dismutase (U/mg protein); GPx, glutathione peroxidase (U/g protein); CAT, catalase (U/g protein); TBARS, thiobarbituric acid reactive substances (mg malondialdehyde/kg meat).

## DISCUSSION

The hydroxychloride mineral source has a crystalline structure formed by covalent bonds between the soluble metal ion, multiple hydroxyl groups, and the chloride ions (Cemin et al., 2017). Besides, the low solubility in water and high solubility in acid solutions (Cao et al., 2000) makes them less reactive with other components in the diet (Olukosi et al., 2018).

In the current study, the pigs fed both minerals from hydroxychloride sources increased by 3.65% and 3.56% in the hot and cold carcass weight, respectively, and also had a greater carcass length than those feds traditional inorganic source, both with high Cu levels. The improvement can explain these results in the ADG (6.59%) and in the final BW (4.70%) associated with reducing diarrhea frequency (23.58%) in the finisher period. Both minerals (Cu and Zn) at a pharmacological level can act as antimicrobial (Pérez et al., 2011), growth promoters (Zhang and Guo, 2007; Coble et al., 2017), and reduce diarrhea (Hill et al., 2000; Zhang and Guo, 2007). Few studies evaluated carcass performance, meat quality, and antioxidant capacity in pigs fed Cu and Zn cosupplemented at a high level from a hydroxychloride source. The effect of minerals varies depending on the age, level and source, immune status, type of ingredients, and interaction of microelements, which may affect other nutrients’ absorption and bioavailability.

The results of the present study are partially consistent with findings of Van Kuijk et al. (2019) that reported based on a meta-analysis that pigs fed 80 mg Zn/kg as hydroxychloride associated with 15 mg Cu/kg have an improvement in lean meat percentage and also at the end of the finisher period, the gain:feed ratio and ADG were both raised by 3.9% compared with those fed sulfates. Villagómez-Estrada et al. (2021) also observed that pigs fed hydroxychloride source showed an improvement by 0.75% carcass yield and tended an increase by 5.94% the ADG at the end of the finisher period than those fed sulfates, regardless of Zn level (20 or 80 mg Zn/kg) associated with 15 mg Cu/kg as CuSO_4_ in all treatments.

On the other hand, no difference was observed in the carcass performance with the providing of Cu and Zn in the form of proteinate amino acid chelate or sulfate associated to level (low, 27 mg Cu/kg and 65 mg Zn/kg and high, 156 mg Cu/kg and 170 mg Zn/kg). Only an improvement in FCR by including proteinate amino acid chelate (Hernández et al., 2008). The Cu and Zn association did not enhance growth performance and carcass characteristics when pigs were fed 125 mg Cu/kg as CuSO_4_ and 150 mg Zn/kg from ZnO (Feldpausch et al., 2016). In broilers, Olukosi et al. (2018) reported that low levels (20 mg Zn/kg), regardless of the sources (sulfate or hydroxychloride), increase to BW in the overall period, and birds also had higher efficiency feed in the grower period when it received hydroxychloride source. Besides, a higher percentage of the breast in carcass yield in the birds fed hydroxychloride and in low level, separately.

The use of Cu and Zn separately in the diet has improved performance and reduced diarrhea in pigs. It does not mean that the animals have experienced deficiency of one of them because the ingredients and the premix can provide these minerals in the diet. We considered it as supplemented only when the authors reported there was an addition of these minerals.

Our results were partially consistent with Coble et al. (2017) research, showing that an additional 150 mg Cu/kg from CuSO_4_ or CHC improved growth performance, increasing hot carcass weight. The growing pigs fed Cu as CuSO_4_ alone or a 50/50 blend of CuSO_4_, and Cu amino acid complex (Cu-AA) showed that a 50/50 blend of Cu optimizes feed efficiency and carcass feed efficiency for pig market weight, regardless of level (70 or 130 mg Cu/kg) (Carpenter et al., 2019). Espinosa et al. (2020) also observed higher ADG in growing pigs fed 150 mg Cu/kg from CHC. On the other hand, Coble et al. (2018) showed that providing 150 mg Cu/kg as a hydroxychloride source associated with by-products in the diet does not influence growth performance. Similar growth performance results were also reported by Zheng et al. (2018) when pigs fed 130 mg Cu/kg as hydroxychloride source.

Most studies suggest that Cu enhances performance during early growing and finishing periods with less or no response during the late finishing period (Hastad et al., 2001). However, in our study and other research, the results were observed at the end of the finishing period, demonstrating that the CHC can be more effective in pig heavier at the market weight (Coble et al., 2017; Villagómez-Estrada et al. 2021).

Zinc also has been used to improve the performance of pigs; the inclusion of this trace mineral in the diet is required for growth performance, metabolic and enzymatic functions, and antioxidant capacity (Kim et al., 2018). It has been shown by Carpenter et al. (2016) that the supplementation of 100 mg Zn/kg as ZnSO_4_ or ZHC maximized BW and ADG. On the other hand, pigs fed 150 mg Zn/kg from hydroxychloride mineral did not improve the growth performance but increased carcass yield percentage (Cemin et al., 2019).

In the present study, the meat quality was similar among pigs fed hydroxychloride or traditional inorganic mineral sources, regardless of level. Pigs fed a low Cu level from hydroxychloride had a trend increase in the loin eye area and reduced cooking loss percentage. This result can be related to higher hot and cold carcass weight observed in these animals. Besides, the cooking loss was positive once it was related to the less water entrapped within the protein structures resulting from the proteins’ denaturation during cooking of pork meat (Hernández-López et al., 2016).

A balanced redox status benefits the livestock and improves meat quality traits, such as water-holding capacity (WHC), color stability, and protection against lipid and protein oxidation, which are essential characteristics consumers show concern about at the time of purchase (Estévez, 2015). The color of meat is related to the total myoglobin content, and the proportion of the myoglobin redox forms oxymyoglobin, deoxymyoglobin, and metmyoglobin. These forms are strongly related to the tissue’s oxidative status influenced by oxygen consumption and the antioxidative capacity (Figueiredo et al., 2008). However, this relation between Mb redox forms and antioxidant enzymes activity was no observed in this study.

In current study, pigs fed 150 mg Cu/kg and zinc from traditional inorganic sources increased SOD activity, with similar GSH-Px, CAT in all treatments. Also, higher lipid oxidation was observed in pigs fed 90 mg Cu/kg and Zn as hydroxychloride sources. The pH 24 h *post-mortem* is an important characteristic and has been reported to influence drip loss, color, and lipid peroxidation. The SOD and GSH-Px activity and T-AOC content were positively correlated to pH 24h *post-mortem* and an opposite trend of MAD (Wang et al., 2018). Pigs fed 130 mg Cu/kg as hydroxychloride source had an increase in the activities of ceruloplasmin and SOD in serum compared with those that received CuSO_4_ on d 30 (Zheng et al., 2018). Nevertheless, we cannot compare it because we evaluate it on the loin muscle.

MDA is the main aldehyde from the lipid decomposition, in this study did not exceed 0.17 mg/kg meat in all treatments. According to Zhang et al. (2016), values up to 0.175 mg MDA/kg are considered very low lipid oxidation levels for pork meat. Perhaps, if the meat samples had been stored for an extended period (12 days), as described by Godziszewska et al. (2017), the lipid oxidation could have been more evident. The degree of the unsaturation in fatty acids, exposure to light and heat, and the presence of molecular oxygen, pro-oxidant and antioxidant components are factors affecting the oxidative stability of lipids (Lima Júnior et al., 2013).

Under physiological conditions, up to 5% of the oxygen reacts to superoxide anions. The CAT and GSH-Px are the major peroxide-removing enzymes in the muscle, while SOD protects against damage by superoxide (Figueiredo et al., 2008; Chen et al., 2010; Chen et al., 2012; Schwarz et al., 2017; Jiao et al., 2018). In turkey meat, an increase of the SOD and GSH-Px enzyme activities has been shown, but without changes in the MDA concentrations, indicating that the tissue’s high antioxidative capacity prevents an apparent increase in lipid oxidation (Janisch et al., 2012).

The diet of animals might alter antioxidants’ levels and activity and the meat storage conditions and pack (Godziszewska et al., 2017). The severity of lipid and protein oxidation in the post-mortem muscle depends on a variety of endogenous mechanisms (free iron, myoglobin and hemoglobin, antioxidant enzymes, lipid content, and composition), and also exogenous factors (exposure to oxygen and light, the temperature of storage and processing) (Soladoye et al., 2015; Domínguez et al., 2019).

## CONCLUSIONS

The high copper content (150 mg Cu/kg) from copper sulfate cosupplemented to zinc oxide impairs the growth performance, carcass and increases diarrhea frequency in grower-finisher pigs and copper and zinc hydroxychloride cosupplementation improves these characteristics.
